# Production of a Novel Protopanaxatriol-Type Ginsenoside by Yeast Cell Factories

**DOI:** 10.3390/bioengineering10040463

**Published:** 2023-04-11

**Authors:** Chen Zhou, Ting Gong, Jingjing Chen, Tianjiao Chen, Jinling Yang, Ping Zhu

**Affiliations:** State Key Laboratory of Bioactive Substance and Function of Natural Medicines, NHC Key Laboratory of Biosynthesis of Natural Products, CAMS Key Laboratory of Enzyme and Biocatalysis of Natural Drugs, Institute of Materia Medica, Chinese Academy of Medical Sciences, Peking Union Medical College, Beijing 100050, China

**Keywords:** protopanaxatriol-type ginsenoside, glycosylation, yeast cell factory, metabolic engineering, synthetic biology

## Abstract

Ginsenosides, the main active compounds in *Panax* species, are glycosides of protopanaxadiol (PPD) or protopanaxatriol (PPT). PPT-type ginsenosides have unique pharmacological activities on the central nervous system and cardiovascular system. As an unnatural ginsenoside, 3,12-Di-*O*-*β*-D-glucopyranosyl-dammar-24-ene-3*β*,6*α*,12*β*,20*S*-tetraol (3*β*,12*β*-Di-*O*-Glc-PPT) can be synthesized through enzymatic reactions but is limited by the expensive substrates and low catalytic efficiency. In the present study, we successfully produced 3*β*,12*β*-Di-*O*-Glc-PPT in *Saccharomyces cerevisiae* with a titer of 7.0 mg/L by expressing protopanaxatriol synthase (PPTS) from *Panax ginseng* and UGT109A1 from *Bacillus subtilis* in PPD-producing yeast. Then, we modified this engineered strain by replacing UGT109A1 with its mutant UGT109A1-K73A, overexpressing the cytochrome P450 reductase ATR2 from *Arabidopsis thaliana* and the key enzymes of UDP-glucose biosynthesis to increase the production of 3*β*,12*β*-Di-*O*-Glc-PPT, although these strategies did not show any positive effect on the yield of 3*β*,12*β*-Di-*O*-Glc-PPT. However, the unnatural ginsenoside 3*β*,12*β*-Di-*O*-Glc-PPT was produced in this study by constructing its biosynthetic pathway in yeast. To the best of our knowledge, this is the first report of producing 3*β*,12*β*-Di-*O*-Glc-PPT through yeast cell factories. Our work provides a viable route for the production of 3*β*,12*β*-Di-*O*-Glc-PPT, which lays a foundation for drug research and development.

## 1. Introduction

*Panax* species have been extensively utilized as traditional medicines in Asia for about 5000 years. Ginsenosides are major components of *Panax* species, which have a wide range of pharmacological effects. Over 150 kinds of natural ginsenosides have been isolated and identified [1,2]. Natural ginsenosides contain glycosyl groups at the C3-OH and/or C20-OH positions of protopanaxadiol (PPD) or at the C6-OH and/or C20-OH positions of protopanaxatriol (PPT), while the glycosylation of dammarenediol-II (DM) at the C3-OH and/or C20-OH positions and the glycosylation of PPD and/or PPT at the C12-OH position lead to the formation of a variety of unnatural ginsenosides. The positions and numbers of hydroxyls and/or glycosyls determine the difference in pharmacological activity among various ginsenosides [3]. PPD-type ginsenosides affect cell cycle distribution and produce cell death signals, showing excellent anti-tumor activity [4,5,6]. Ginsenoside Rg3 has been used in clinical applications for twenty years and shows inhibitory effects on lung cancer and liver cancer [7]. Ginsenoside compound K has been proven to have a range of anti-cancer activities against lung cancer, leukemia, breast cancer, and colon cancer [8,9,10,11]. Ginsenoside Rh2 shows anti-cancer activity against lung cancer and liver cancer by mediating the apoptosis of cancer cells and suppressing cell proliferation and migration [12,13]. PPT-type ginsenosides have unique activities on the central nervous system and cardiovascular system. PPT, ginsenoside Rh1, and Rg1 can enhance the excitability of the hippocampus, which is conducive to the acquisition, consolidation, and reproduction of learning and memory [14,15,16,17]. Rg1 is also the most promising lead compound for anti-aging due to its effect on clearing free radicals. Ginsenoside Rb1 and Rg2 can effectively prevent or slow the development of Alzheimer’s disease [18]. Rg2 directly acts on human neuron nicotinic acetylcholine receptors (nAChRs) and regulates their desensitization [19]. Ginsenoside Re exhibits anti-peripheral nerve injury, anti-cerebral ischemia, and anti-neurotoxicity activities on nervous system diseases [20,21,22].

**Figure 1:**
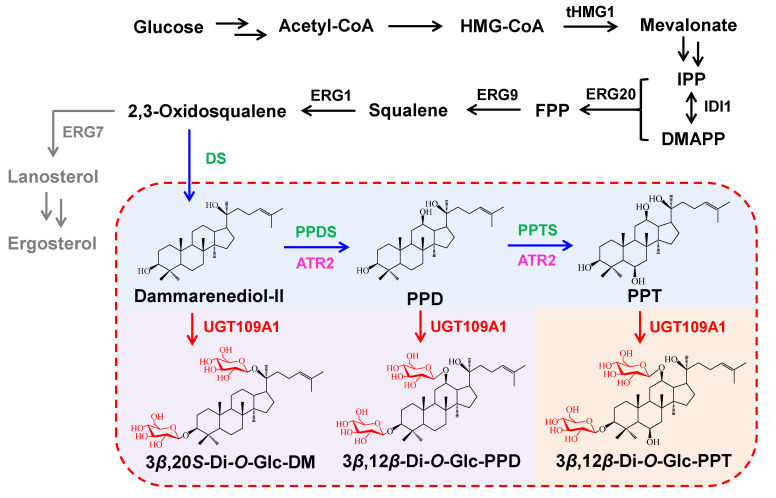
Construction of the biosynthetic pathway of 3*β*,12*β*-Di-*O*-Glc-PPT in the engineered yeast. The intrinsic *Saccharomyces cerevisiae* pathway is indicated by black and grey arrows. The double arrows show the multiple-step reactions. The *Panax ginseng* pathway is shown by blue arrows; the glycosylation reactions are shown by red arrows. The genes in black are up-regulated; the gene in grey is down-regulated; the genes in green are from *P. ginseng*; the gene in magenta is from *Arabidopsis thaliana*; and the gene in red is from *Bacillus subtilis*. The compounds in blue background are aglycones; the compounds in purple background are by-products; and the compound in orange background is target product.

Unnatural ginsenosides may show unexpected activities compared with the natural ones produced in *Panax* species. Ginsenoside Ia was transformed from F1 by BSGT1 of *Bacillus subtilis* and showed an inhibitory effect on melanogenesis in B16BL6 cells [23]. In our previous study, a new UDP-glycosyltransferase (UGT) named UGT109A1 from *B. subtilis* was demonstrated to catalyze the glycosylation of DM at the C3-OH and C20-OH positions and PPD at the C3-OH and C12-OH positions, respectively, leading to the formation of a series of unnatural ginsenosides [24]. It has been confirmed that the anti-lung cancer activity of 3,12-Di-*O-β*-D-glucopyranosyl-dammar-24-ene-3*β*,12*β*,20*S*-triol (3*β*,12*β*-Di-*O*-Glc-PPD) is superior to that of the natural ginsenoside Rg3 [24]; 3,20-Di-*O*-*β*-D-glucopyranosyl-dammar-24-ene-3*β*,20*S*-diol (3*β*,20*S*-Di-*O*-Glc-DM) displays better anti-pancreatic activity than both of the natural ginsenosides Rg3 and F2 [25]; and 3-*O*-*β*-D-glucopyranosyl-dammar-24-ene-3*β*,20*S*-diol (3*β*-*O*-Glc-DM) exhibits a higher activity on colon cancer than that of Rg3 and compound K [26]. UGT109A1 also can glycosylate the C3-OH and C12-OH positions of PPT simultaneously to produce another unnatural ginsenoside, 3*β*,12*β*-Di-*O*-Glc-PPT (Figure 1). Due to the presence of the glucosyl moiety at the C12 position, it is speculated that 3*β*,12*β*-Di-*O*-Glc-PPT may have unique pharmacological activity different from natural PPT-type ginsenosides. Thus, it is necessary to accumulate a large amount of 3*β*,12*β*-Di-*O*-Glc-PPT for research on its pharmacological activity.

At present, there are many ways to produce ginsenosides, including chemical synthesis, enzymatic catalysis, biotransformation, and hetero-biosynthesis. Chemical synthesis has disadvantages in terms of sustainability, selectivity, and renewability. The methods of enzymatic catalysis and biotransformation are becoming more and more mature, but they still need a large amount of expensive substrates, which is a bottleneck for their development and utilization. The development of synthetic biology has made it possible to achieve the de novo biosynthesis of ginsenosides, which has become a new research hotspot. *Saccharomyces cerevisiae* has an intrinsic mevalonate biosynthesis pathway, providing the basic precursor of 2,3-oxidosqualene for triterpene synthesis. It also produces UDP-glucose (UDPG) and is therefore considered as an ideal chassis for constructing cell factories of triterpenoid saponins. Great progress has been made in the synthesis of PPD-type ginsenosides from cheap carbon sources in *S. cerevisiae*, such as Rg3 (1.3 mg/L) [27], Rh2 (2.25 g/L) [28], and compound K (5.74 g/L) [29]. The biosynthetic pathways of PPT-type ginsenosides are longer than those of PPD-type ginsenosides. The low efficiency of protopanaxatriol synthase (PPTS) makes the heterologous biosynthesis of PPT-type ginsenosides more difficult. Dai et al. [30] constructed the biosynthetic pathway of PPT in yeast by introducing *Panax ginseng* dammarenediol-II synthase (DS), protopanaxadiol synthase (PPDS), PPTS, and *Arabidopsis thaliana* ATR1 and overexpressing the key enzymes involved in the upstream pathway to improve the precursor supply. The PPT titer of the engineered yeast was 15.9 mg/L. Li et al. [31] constructed a high-yield PPT-producing yeast strain through protein engineering with a titer of over 5 g/L in a 1.3 L bioreactor. By introducing PgUGT71A54, the cell factory producing ginsenoside Rg1 was built with a titer of 1.95 g/L.

In this study, we utilized the glycosyltransferase UGT109A1 from *B. subtilis* to achieve the production of 3*β*,12*β*-Di-*O*-Glc-PPT in the engineered yeast. First, we designed the co-expressed gene module harboring PPTS and UGT109A1, which was integrated into the chromosomes of the PPD-producing yeast, resulting in the 3*β*,12*β*-Di-*O*-Glc-PPT-producing strain with a titer of 7.0 mg/L. Then, we replaced UGT109A1 with its mutant UGT109A1-K73A to enhance the glycosylation efficiency. We also modified the engineered strain by overexpressing the cytochrome P450 reductase ATR2 and/or overexpressing the key enzymes of UDPG biosynthesis to improve the supply of the glycosyl acceptor and donor. Unexpectedly, the application of these optimization strategies did not increase the 3*β*,12*β*-Di-*O*-Glc-PPT production. However, an unnatural PPT-type ginsenoside, 3*β*,12*β*-Di-*O*-Glc-PPT, was obtained by the yeast cell factories, providing a raw material for innovative drug research.

## 2. Materials and Methods

### 2.1. Strains

*Escherichia coli* Trans1-T1 (TransGen Biotech, Beijing, China) was used for plasmid amplification. The PPD-producing strain YPU [32] was chosen as the yeast chassis for constructing the engineered yeasts.

### 2.2. The Engineered Yeast Construction

The gene expression cassettes harboring *ATR2*, *UGT109A1*, *UGT109A1-K73A*, *PGM1*, *PGM2,* and *UGP1* were constructed according to the method described previously [24,25,26,32]. The gene of *PPTS* was synthesized (GenScript, Nanjing, China) according to the codon bias of *S. cerevisiae* and was inserted into gene expression cassette with promoter *TEF1* and terminator *CYC1*.

The integration modules containing *PPTS*, *UGT109A1,* and *HIS3* were transformed into the *δ1* site of the PPD-producing strain YPU to generate strain YPUT. The integration modules containing *PPTS*, *UGT109A1-K73A,* and *HIS3* were transformed into the *δ1* site of strain YPU to generate strain YPUK. The integration modules containing *PPTS*, *ATR2*, *UGT109A1,* and *HIS3* were transformed into the *δ1* site of strain YPU to generate strain YPUT-B. The integration modules containing *PGM1*, *PGM2*, *UGP1*, *PPTS*, *UGT109A1,* and *HIS3* were transformed into the *δ1* site of strain YPU to construct strain YPUT-C. The integration modules harboring *PGM1*, *PGM2*, *UGP1*, *PPTS*, *ATR2*, *UGT109A1,* and *HIS3* were transformed into the *δ1* site of strain YPU to construct strain YPUT-D. Colonies of each group were randomly picked from SD-HIS-LEU-TRP-URA plates and validated by PCR amplification. Eight correct colonies were cultivated in YPD medium for 6 days and the titers of 3*β*,12*β*-Di-*O*-Glc-PPT were detected.

All the constructed strains are listed in Table 1.

### 2.3. Fermentation and Product Extraction of the Engineered Yeasts

The shake flask fermentation of all the engineered strains was performed as described in our previous work [32]. When the fermentation was finished, the cultured cells were harvested by centrifugation (10,000× *g*, 3 min). The extraction and analysis methods of ginsenosides and aglycones have been described previously [32]. All data are presented as means ± SD of three independent repeat experiments (*n* = 3).

### 2.4. Product Analysis by HPLC, HPLC-ESI-MS and NMR

The methods of HPLC, HPLC-ESI-MS, and NMR were used as previously reported [24]. The detailed conditions of analytic and semi-preparative HPLC for extracts of yeast fermentation are described in Table S1.

## 3. Results

### 3.1. Constructing 3β,12β-Di-O-Glc-PPT-Producing Yeast

We have demonstrated that UGT109A1 from *B. subtilis* can transfer a glucosyl moiety to the C3-OH and C12-OH positions of PPT simultaneously to yield 3*β*,12*β*-Di-*O*-Glc-PPT [24]. The recombinant enzyme can catalyze the formation of 3*β*,12*β*-Di-*O*-Glc-PPT by using UDPG as a sugar donor and PPT as a sugar acceptor in a reaction system. Since PPT is a very expensive chemical, we sought to construct engineered yeasts to achieve the de novo biosynthesis of 3*β*,12*β*-Di-*O*-Glc-PPT, which omits the external supplementary of PPT (Figure 1).

**Figure 2:**
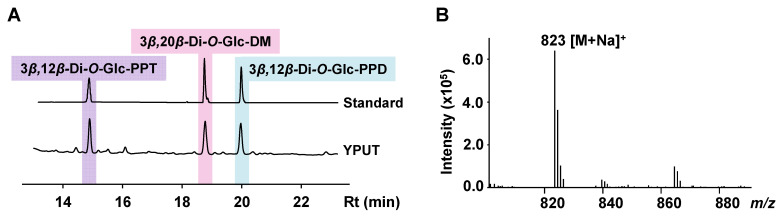
(**A**) HPLC detection of the *n*-butanol extracts of strain YPUT. (**B**) ESI-MS spectrum of 3*β*,12*β*-Di-*O*-Glc-PPT.

Previously, we constructed a PPD-producing strain YPU that could produce 93.1 mg/L PPD [32]. For producing 3*β*,12*β*-Di-*O*-Glc-PPT, the gene expression module containing *PPTS* from *P. ginseng* and *UGT109A1* from *B. subtilis* was inserted into the *δ1* site of strain YPU, generating strain YPUT. After cultivation for six days, the target product 3*β*,12*β*-Di-*O*-Glc-PPT in the intracellular extracts was identified by an HPLC analysis (Figure 2A), and its structure was elucidated by HPLC-ESI-MS, ^1^H NMR, and ^13^C NMR (Figure 2B and Figure S1). The titer of 3*β*,12*β*-Di-*O*-Glc-PPT in strain YPUT was 7.0 mg/L. There was no PPT detected from the extract of YPUT, but there was a large amount of 3*β*,12*β*-Di-*O*-Glc-PPD and 3*β*,20*S*-Di-*O*-Glc-DM (Figure 2A). It suggested that the low-efficiency PPTS was a key limiting factor in the biosynthetic pathway of 3*β*,12*β*-Di-*O*-Glc-PPT. In addition, neither 3*β*-*O*-Glc-PPT nor 12*β*-*O*-Glc-PPT was detected in the extract. A possible reason is that UGT109A1 can effectively and rapidly transform 3*β*-*O*-Glc-PPT or 12*β*-*O*-Glc-PPT into 3*β*,12*β*-Di-*O*-Glc-PPT. Further experiments are needed to determine the order in which the C3-OH and C12-OH of PPT are glycosylated. To the best of our knowledge, this is the first report of the de novo biosynthesis of 3*β*,12*β*-Di-*O*-Glc-PPT in yeast through the strategy of metabolic engineering.

### 3.2. Improving the Catalytic Efficiency of UGT109A1 to Promote the Glycosylation of PPT

Although we obtained the 3*β*,12*β*-Di-*O*-Glc-PPT-producing yeast, the yield was low. The catalytic efficiency and expression level of biosynthetic enzymes have important effects on the yield of the target products. The K73A mutation of UGT109A1 has been proven to markedly improve 3*β*,12*β*-Di-*O*-Glc-PPD production because the mutation expands the active pocket and increases its hydrophobicity [32]. Since the structures of 3*β*,12*β*-Di-*O*-Glc-PPD and 3*β*,12*β*-Di-*O*-Glc-PPT differ only at the C6 position, at which 3*β*,12*β*-Di-*O*-Glc-PPT possesses an extra hydroxyl group, we supposed that the mutant UGT109A1-K73A might also improve the glycosylation efficiency towards PPT and promote the yield of 3*β*,12*β*-Di-*O*-Glc-PPT. Strain YPUK was therefore constructed by replacing the wild-type UGT109A1 with its mutant UGT109A1-K73A.

**Figure 3:**
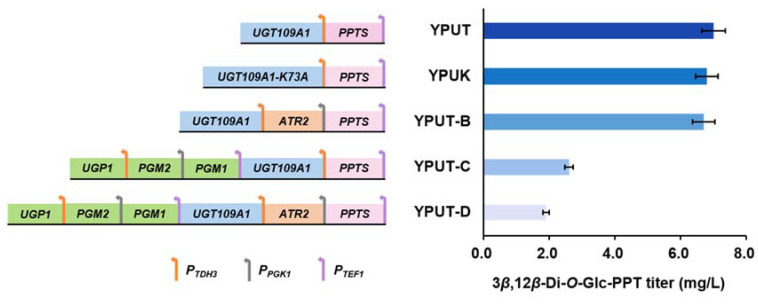
Production of 3*β*,12*β*-Di-*O*-Glc-PPT in the engineered strains. The gene in pink box is derived from *P. ginseng*. The gene in orange box is derived from *A. thaliana*. The gene in blue box is derived from *B. subtilis*. The genes in green boxes are derived from *S. cerevisiae*.

After cultivation for six days, the titer of 3*β*,12*β*-Di-*O*-Glc-PPT was 6.8 mg/L in strain YPUK, which was even slightly lower than that in strain YPUT (Figure 3). There were two possible reasons for this result: one is that the K73A mutation did not improve the glycosylation efficiency of UGT109A1 towards PPT and even inhibited it to a certain extent; the other is that the catalytic efficiency of UGT109A1 was improved, but the low efficiency of PPTS limited the supply of precursor PPT. Notably, PPT was undetected in either strain YPUT or strain YPUK, indicating that our second conjecture was more likely. Thus, we speculated that the key was to ensure the sufficient supply of the precursor in order to boost the production of 3*β*,12*β*-Di-*O*-Glc-PPT.

### 3.3. Improving Precursor Supply for the Production of 3β,12β-Di-O-Glc-PPT

The supply of both glycosyl receptor PPT and glycosyl donor UDPG is important to the production of PPT-type ginsenosides. It has been reported that the balance between CYP450s and their reductases has a significant role in the formation of natural products [33]. In the host strain YPU, the reductase ATR2 from *A. thaliana* was shared by PPDS and PPTS [30]. Herein, we hypothesize that the low 3*β*,12*β*-Di-*O*-Glc-PPT production may be due to the mismatch of the expression levels of PPTS and ATR2. Thus, we optimized the oxidation–reduction system by overexpressing ATR2 in *S. cerevisiae*. The gene module harboring PPTS, ATR2, and UGT109A1 was inserted into the *δ1* site of strain YPU, resulting in strain YPUT-B.

UDPG is an important sugar donor, and its natural supply is very limited in *S. cerevisiae*, and thus it probably cannot meet the demand for the synthesis of ginsenosides. Wang et al. [34] overexpressed *S. cerevisiae* phosphoglucomutase 1 (PGM1), phosphoglucomutase 2 (PGM2), and UDPG pyrophosphorylase 1 (UGP1) in the engineered yeast, increasing the production of UDPG more than eight times. Improving the supply of UDPG in yeast has effectively promoted the production of ginsenosides F1 and compound K in recombinant strains [34,35]. Here, we generated the expression cassettes of the three enzymes and integrated them into the *δ1* site of strain YPU together with those of PPTS and UGT109A1, resulting in strain YPUT-C. Then, we overexpressed ATR2 and the key enzymes involved in UDPG synthesis to increase the supply of both the glycosyl donor and receptor at the same time, resulting in strain YPUT-D.

After cultivation for six days, the production of 3*β*,12*β*-Di-*O*-Glc-PPT in these engineered strains was identified and quantified. The 3*β*,12*β*-Di-*O*-Glc-PPT titers of the three strains were all lower than that of YPUT (Figure 3). The lowest titer of 3*β*,12*β*-Di-*O*-Glc-PPT appeared in strain YPUT-D (1.9 mg/L) (Table 2), which was only 27% that in strain YPUT. According to the results, the optimization of the glycosyl donor and receptor supply did not improve the production of 3*β*,12*β*-Di-*O*-Glc-PPT in the engineered strain. What is more, the overexpression of enzymes related to the synthesis of UDPG showed an inexplicable inhibitory impact on the production of 3*β*,12*β*-Di-*O*-Glc-PPT.

## 4. Discussion

As the major active components of *Panax* species, ginsenosides exhibit many pharmacological activities [36,37,38,39,40,41,42,43,44]. The different positions, numbers, and types of sugar moieties result in the different medicinal properties of ginsenosides. UGT109A1 from *B. subtilis* can glycosylate triterpenoid aglycones DM, PPD, and PPT to yield a variety of unnatural ginsenosides [24]. Among them, 3*β*,12*β*-Di-*O*-Glc-PPT is a novel compound reported by our lab. However, it remains ambiguous whether UGT109A1 can synthesize 3*β*,12*β*-Di-*O*-Glc-PPT in vivo in *S. cerevisiae*. In the present work, we integrated *PPTS* and *UGT109A1* genes into the chromosome of the PPD-producing strain, and 3*β*,12*β*-Di-*O*-Glc-PPT was detected as expected, indicating that PPT was synthesized by PPTS and then glycosylated by UGT109A1 in vivo.

The catalytic efficiency of UGT109A1-K73A towards PPD was improved markedly compared with that of the wild-type UGT109A1 [32]. Considering the similarity of the structures of PPD and PPT, strain YPUK was constructed by expressing the mutant UGT109A1-K73A to improve the glycosylation efficiency. However, the titer of 3*β*,12*β*-Di-*O*-Glc-PPT in strain YPUK was not improved compared with that in strain YPUT. Since the distance between the C6-OH and C3-OH of 3*β*,12*β*-Di-*O*-Glc-PPT was close, the C6-OH may change the relative position of the substrate in the binding pocket of UGT109A1-K73A and hinder the glycosylation process. Moreover, the aglycone PPT was undetectable in the two engineered strains. It can be speculated that the catalytic efficiency of PPTS is far less than the glycosylation efficiency of UGT109A1, so the rate of PPT biosynthesis cannot keep up with the rate of glycosylation to form 3*β*,12*β*-Di-*O*-Glc-PPT.

The precursor supply level is known to play a crucial role in improving the production of the target products in the engineered strains. Since ATR2 derived from *A. thaliana* was considered to be a universal NADPH-cytochrome P450 reductase (CPR) shared by PPDS and PPTS [30], PPTS was effectively expressed in strain YPUT. The 3*β*,12*β*-Di-*O*-Glc-PPT titer of strain YPUT-B was lower than that of strain YPUT, implying that the overexpression of ATR2 in strain YPUT-B may destroy the balance between PPTS and its reductase partner. In the meantime, PPT was undetected in either strain YPUT or YPUT-B. PPTS turned out to be a rate-limiting enzyme for the production of PPT-type ginsenosides. Redox partners play an important role for CYP450s. Different CPRs have diverse effects on the catalytic activity of CYP450s. An appropriate CPR can promote the efficient synthesis of compounds. CYP450/CPR pairs excavated from the same plant species generally show better effects than unnatural combinations [45,46,47,48]. RoCPR1 from *Rosmarinus officinalis* stood out in a group of plant-derived CPRs when co-expressed with the RoCYP01 from *R. officinalis* to promote the production of betulinic acid (BA). The titer of BA obtained from the engineered strain increased to more than 1 g/L [49]. Zhu et al. [50] compared the coupling efficiency of six CPRs from different plants, including *A. thaliana*, *Lotus japonicus*, *G. uralensis,* and *Medicago sativa*, with Uni25647 and CYP72A154 from *Glycyrrhiza uralensis*. The results showed that GuCPR1 from *G. uralensis* exhibited the best coupling efficiency with the two CYP450s among the six CPRs. However, Wang et al. [35] found that the CPR from *Vitis vinifera* (VvCPR) combined with PgPPDS boosted the PPD production better than those from *A. thaliana* and *P. ginseng*. Since the CPR for PPTS with a high efficiency has not yet been identified, more CPRs from different sources should be investigated to improve the production of PPT.

In addition, there is a UDPG biosynthetic pathway composed of PGM1, PGM2, and UGP1 in *S. cerevisiae*, but its natural supply cannot meet the demand for the synthesis of ginsenosides. Previously, we improved the production of rare ginsenoside F2 and unnatural ginsenoside 3*β*,20*S*-Di-*O*-Glc-DM in yeast successfully by overexpressing the key enzymes related to the biosynthesis of UDPG [25]. In the present study, these enzymes were also overexpressed in an attempt to improve the yield of 3*β*,12*β*-Di-*O*-Glc-PPT. As shown in Table 2, the titer of 3*β*,12*β*-Di-*O*-Glc-PPT in strain YPUT-C and strain YPUT-D decreased markedly compared with that of strain YPUT, while the titer of 3*β*,12*β*-Di-*O*-Glc-PPD increased from 4.8 to 7.2 mg/L. These results suggested that there was competition between the glycosylation of PPD and PPT. Similar studies showed that amplifying the supply of UDPG apparently increased the production PPD-type ginsenosides [34,35]. Since the catalytic activity of UGT109A1 towards PPD was higher than that towards PPT, the optimization of the UDPG supply was more effective in improving the glycosylation of PPD. The production of 3*β*,12*β*-Di-*O*-Glc-PPT inevitably decreased in the engineered strains. 

Other strategies need to be further developed to increase the yield of 3*β*,12*β*-Di-*O*-Glc-PPT in the engineered yeast, including the protein engineering of PgPPTS and UGT109A1, regulation of key enzymes’ expression, utilization of subcellular compartments, and cofactor engineering. Moreover, the yield of 3*β*,12*β*-Di-*O*-Glc-PPT can be further increased by optimizing the fermentation conditions of the engineered strain.

## 5. Conclusions

In this study, we achieved the de novo biosynthesis of 3*β*,12*β*-Di-*O*-Glc-PPT in *S. cerevisiae* by expressing PPTS and UGT109A1 in the PPD-producing strain, and the titer was 7.0 mg/L. Then, we sought to improve the efficiency of glycosyltransferase by replacing the wild-type UGT109A1 with its mutant UGT109A1-K73A and to increase the supply of the glycosyl receptor and donor by overexpressing ATR2 and the enzymes involved in the biosynthetic pathway of UDPG. Unexpectedly, these strategies did not boost the titer of 3*β*,12*β*-Di-*O*-Glc-PPT. However, to the best of our knowledge, this is the first report of developing a yeast cell factory for the de novo biosynthesis of unnatural ginsenoside 3*β*,12*β*-Di-*O*-Glc-PPT. This study has explored a green and sustainable approach to producing 3*β*,12*β*-Di-*O*-Glc-PPT based on synthetic biology, which lays a foundation for research on its pharmacological activity.

## Figures and Tables

**Table 1:** The strains used in this study.

Strain	Genotype or Characteristic	Target Product
YPU	*P_TEF1_-synDS-GFP-T_CYC1_*, *P_TDH3_-tPPDS-L18I-T_ADH2_*, *P_TEF2_-ATR2-T_TPI1_*, *P_PGK1_-tHMG1-T_ADH1_*, *P_TEF1_-HAC1-T_CYC1_* and *TRP1* marker gene integrated into the *rDNA* site, and *P_TDH3_-IDI1-T_TPI1_*, *P_PGK1_-ERG20-T_ADH1_*, *P_TEF1_-ERG9-T_CYC1_*, *P_PGK1_-ERG1-T_ADH1_*, *P_TEF1_-ERG7*^−^*-T_CYC1_* and *LEU2* marker gene integrated into the *δ4* site of Y-ΔHXK2	PPD
YPUT	*P_TEF1_-PPTS-T_CYC1_*, *P_TDH3_-UGT109A1-T_ADH2_* and *HIS3* marker gene integrated into the *δ1* site of YPU	3*β*,12*β*-Di-*O*-Glc-PPT
YPUK	*P_TEF1_-PPTS-T_CYC1_*, *P_TDH3_-UGT109A1-K73A-T_ADH2_* and *HIS3* marker gene integrated into the *δ1* site of YPU	3*β*,12*β*-Di-*O*-Glc-PPT
YPUT-B	*P_TEF1_-PPTS-T_CYC1_*, *P_PGK1_-ATR2-T_TPI1_*, *P_TDH3_-UGT109A1-T_ADH2_* and *HIS3* marker gene integrated into the *δ1* site of YPU	3*β*,12*β*-Di-*O*-Glc-PPT
YPUT-C	*P_TEF1_-PGM1-T_CYC1_*, *P_PGK1_-PGM2-T_ADH1_*, *P_TDH3_-UGP1-T_ADH2_*, *P_TEF1_-PPTS-T_CYC1_*, *P_TDH3_-UGT109A1-T_ADH2_* and *HIS3* marker gene integrated into the *δ1* site of YPU	3*β*,12*β*-Di-*O*-Glc-PPT
YPUT-D	*P_TEF1_-PGM1-T_CYC1_*, *P_PGK1_-PGM2-T_ADH1_*, *P_TDH3_-UGP1-T_ADH2_*, *P_TEF1_-PPTS-T_CYC1_*, *P_PGK1_-ATR2-T_TPI1_*, *P_TDH3_-UGT109A1-T_ADH2_* and *HIS3* marker gene integrated into the *δ1* site of YPU	3*β*,12*β*-Di-*O*-Glc-PPT

**Table 2:** Yields of 3*β*,12*β*-Di-*O*-Glc-PPT and other by-products produced in the engineered strains.

Yield ^a^ (mg/L)	YPUT	YPUT-B	YPUT-C	YPUT-D
3*β*,12*β*-Di-*O*-Glc-PPT	7.0 ± 0.3	6.7 ± 0.4	2.6 ± 0.1	1.9 ± 0.4
3*β*,12*β*-Di-*O*-Glc-PPD	4.8 ± 0.2	5.2 ± 0.5	6.3 ± 0.5	7.2 ± 0.4
3*β*,20*S*-Di-*O*-Glc-DM	3.8 ± 0.2	3.6 ± 0.2	3.7 ± 0.2	3.1 ± 0.3
PPT	0	0	0	0

^a^ Yield referred to the sum of intracellular and extracellular contents.

## Data Availability

Not applicable.

## Supplementary Materials

The following supporting information can be downloaded at: https://www.mdpi.com/article/10.3390/bioengineering10040463/s1, Figure S1: The ^1^H-NMR (A) and ^13^C-NMR (B) spectra of 3,12-Di-*O*-*β*-D-glucopyranosyl-dammar-24-ene-3*β*,6*α*,12*β*,20*S*-tetraol (3*β*,12*β*-Di-*O*-Glc-PPT) extracted from strain YPUT; Table S1: The HPLC conditions for analysis and preparation of products from the engineered strains.
