# Supplementary material for: Production of a Novel Protopanaxatriol-Type Ginsenoside by Yeast Cell Factories
Source: Bioengineering (Basel). 2023 Apr 11;10(4):463. doi: 10.3390/bioengineering10040463 (PMC10135449; doi:10.3390/bioengineering10040463)
Supplement: Supplementary file 1 [file bioengineering-10-00463-s001.zip › bioengineering-2274152-supplementary.pdf]

Supplementary Information

Figure S1. The ^{1}H-NMR (**A**) and ^{13}C-NMR (**B**) spectra of 3,12-Di-*O*-*β*-D-glucopyranosyl-dammar-24-ene-3*β*,6*α*,12*β*,20*S*-tetraol (3*β*,12*β*-Di-*O*-Glc-PPT) extracted from strain YPUT.

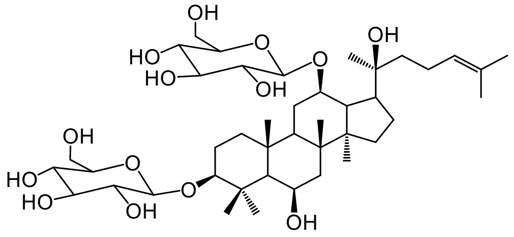

3*β*,12*β*-Di-*O*-Glc-PPT

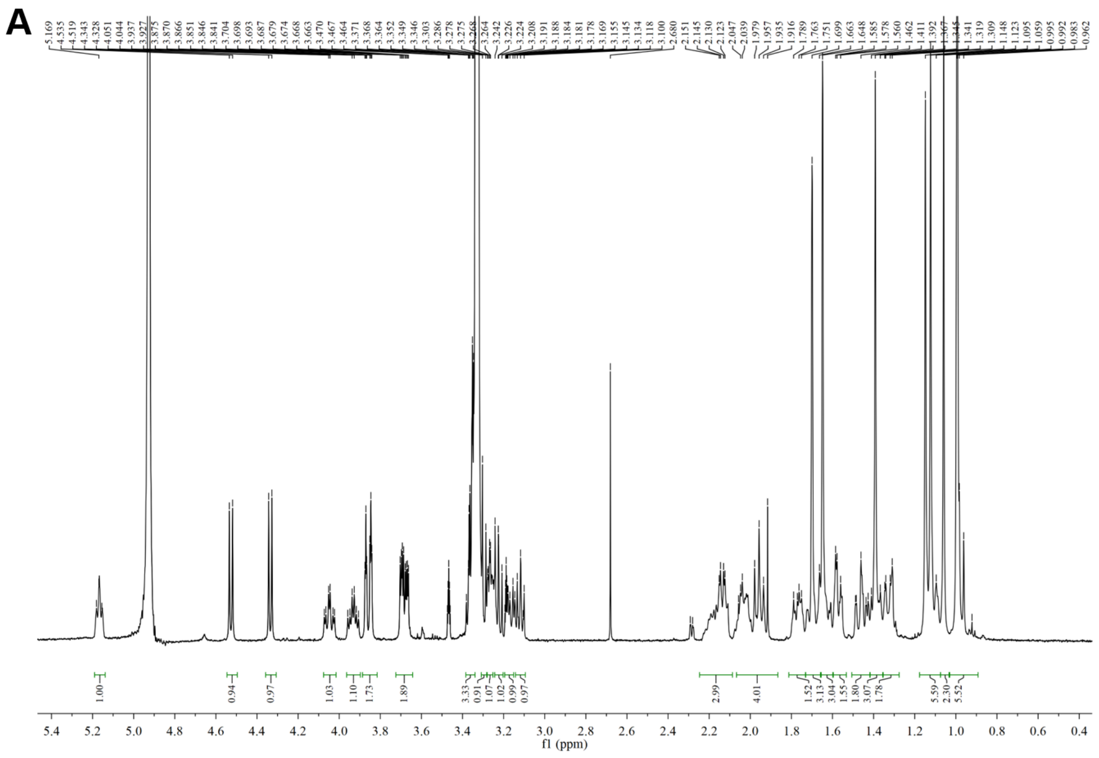

1H-NMR (500MHz, in Methanol-d_4) δ 5.15 (1H, t, J = 6Hz, H-24), 4.52 (1H, d, J =8Hz, H-1″), 4.33 (1H, d, J = 7.5Hz, H-1′), 4.02 (1H, td, J = 11Hz, 5Hz, H-6), 3.14 (1H, d, J = 5Hz, H-3), 3.10 (1H, m, H-12), 1.70 (3H, s, H-28), 1.65 (3H, s, H-26), 1.39 (3H, s, H-27), 1.15 (3H, s, H-29), 1.12 (3H, s, H-21), 1.06 (3H, s, H-19), 1.00 (6H, s, H-30/H-18).

B

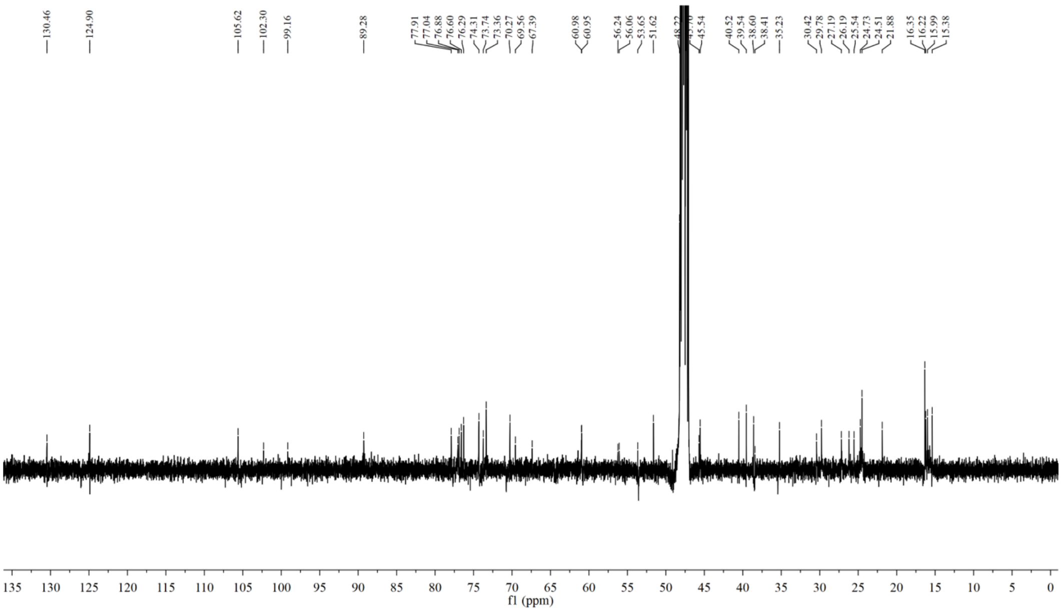

^{13}C-NMR (125 MHz, Methanol-d_4) δ 130.46 (C-25), 124.90 (C-24), 89.28 (C-3), 77.91 (C-12), 73.36 (C-20), 67.39 (C-6), 60.98 (C-5), 53.63 (C-17), 51.62 (C-14), 48.22 (C-9), 45.57 (C-13), 45.54 (C-7), 40.52 (C-8), 39.54 (C-4), 38.60 (C-10), 38.41 (C-1), 35.23 (C-22), 30.42 (C-11), 29.78 (C-28), 27.19 (C-15), 26.19 (C-2), 25.54 (C-21), 24.73 (C-16), 24.51 (C-26), 21.88 (C-23), 16.35 (C-18 and C-27), 16.22 (C-19), 15.99 (C-30), 15.38 (C-29), 105.62 (C-1′), 73.74 (C-2′), 76.88 (C-3′), 70.27 (C-4′), 76.60 (C-5′), 60.95 (C-6′), 99.16 (C-1″), 74.31 (C-2″), 76.29 (C-3″), 69.56 (C-4″), 77.04 (C-5″), 60.98 (C-6″).

Table S1. The HPLC conditions for analysis and preparation of products from the engineered strains.

Method	Gradient	Product
1	0 min, 10% acetonitrile; 20 min, 55% acetonitrile; 30 min, 70% acetonitrile; 31 min, 100% acetonitrile; 40 min, 100% acetonitrile; 41 min, 10% acetonitrile; 50 min, 10% acetonitrile	3*β*,20*S*-Di-*O*-Glc-DM<br>3*β*,12*β*-Di-*O*-Glc-PPD<br>3*β*,12*β*-Di-*O*-Glc-PPT<br>PPT
2	0 min, 20% acetonitrile; 20 min, 85% acetonitrile; 30 min, 100% acetonitrile; 40 min, 100% acetonitrile; 41 min, 20% acetonitrile; 50 min, 20% acetonitrile	3*β*,20*S*-Di-*O*-Glc-DM<br>3*β*,12*β*-Di-*O*-Glc-PPD<br>PPD<br>DM
